# Combined Reconstruction of the Medial Patellofemoral Ligament and the Medial Quadriceps Tendon–Femoral Ligament Using Rectus Femoris Tendon Autograft

**DOI:** 10.1002/atn2.70002

**Published:** 2026-04-29

**Authors:** Lokman Kehribar, John P. Fulkerson, Ahmet Emin Okutan

**Affiliations:** ^1^ School of Medicine, Department of Orthopaedic Surgery Dokuz Eylül University İzmir Turkey; ^2^ School of Medicine, Department of Orthopaedics and Rehabilitation Yale University New Haven Connecticut U.S.A.; ^3^ School of Medicine, Department of Orthopaedic Surgery Samsun University Samsun Turkey

## Abstract

Patellofemoral instability remains a challenging condition, especially in skeletally immature patients, who are at increased risk of recurrence. The medial patellofemoral ligament has historically been the focus of surgical reconstruction; however, recent anatomic studies have also highlighted the medial quadriceps tendon–femoral ligament as part of the medial patellofemoral complex, a key stabilizer during early knee flexion. Recognition of the medial patellofemoral complex has prompted a shift toward combined reconstruction techniques that more accurately replicate native anatomy. The rectus femoris tendon autograft, recently applied in anterior cruciate ligament surgery, offers unique advantages owing to its preserved patellar attachment. In this Technical Note, we describe an anatomic V‐shaped reconstruction of the medial patellofemoral complex, combining both medial patellofemoral ligament and the medial quadriceps tendon–femoral ligament with a rectus femoris autograft, providing a biomechanically rational approach that restores physiologic stability while minimizing the risk of patellar fracture.

## Graphical abstract

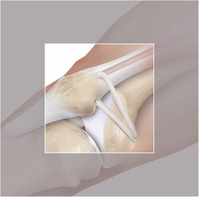

**VIDEO 1:** Surgical steps of the right knee in the supine position, demonstrating the technique for combined reconstruction of the medial patellofemoral ligament (MPFL) and the medial quadriceps tendon‐femoral ligament (MQTFL) using a rectus femoris tendon autograft with preserved patellar insertion. The video illustrates patient positioning, graft harvest and preparation, femoral tunnel creation, graft rerouting, and fixation, highlighting the formation of a V‐shaped medial patellofemoral complex (MPFC) to restore patellar stability. Video content can be viewed at https://doi.org/10.1002/atn2.70002.

Patellofemoral instability is a common and challenging condition, particularly in skeletally immature patients, who show more than twice the risk of recurrent dislocation compared with skeletally mature individuals.^1^, ^2^, ^3^ Historically, surgical treatment has centered on reconstruction of the medial patellofemoral ligament (MPFL), given its role as the primary restraint to lateral patellar translation during early knee flexion.^4^ Recently, anatomical studies have identified an additional component of the proximal medial patellar restraints, the medial quadriceps tendon–femoral ligament (MQTFL), which shares the same femoral origin as the MPFL but inserts on the quadriceps tendon.^5^, ^6^ Together, these structures constitute the medial patellofemoral complex (MPFC), a critical stabilizer of the patella during early knee flexion.^7^ Over the past decade, increasing recognition of the MPFC has shifted surgical strategies toward techniques that more closely replicate native anatomy.^7^, ^8^

Recently, the rectus femoris (RF) tendon autograft has emerged as a promising graft choice in anterior cruciate ligament (ACL) surgery.^9^, ^10^ In this Technical Note, we describe an anatomic reconstruction of the MPFC, combining both the MPFL and MQTFL, using an RF tendon autograft. Owing to its native patellar attachment, rerouting the RF tendon to function as part of the MPFC may represent a biomechanically rational and clinically valuable strategy to restore patellofemoral stability while minimizing the risk of patellar fracture.

## SURGICAL TECHNIQUE

**FIGURE 1:**
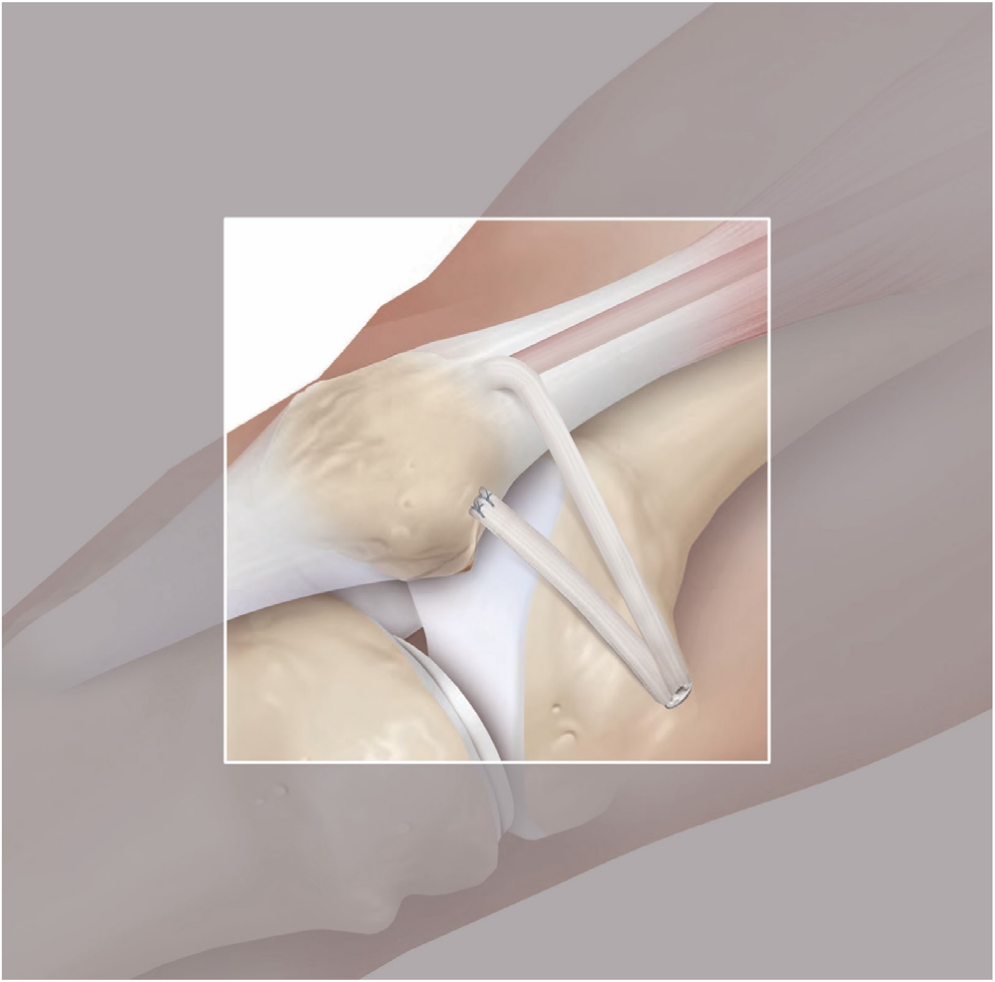
An overview of the surgical procedure illustrating the rerouting of the rectus femoris tendon autograft and its orientation for the reconstruction of both the medial quadriceps tendon–femoral ligament and the medial patellofemoral ligament.

A detailed video of the technique is shown in Video 1. An overview of the surgical procedure is illustrated in Figure 1, highlighting the rerouting of the RF tendon autograft and the appropriate graft orientation to reconstruct both the MQTFL and MPFL.

### Patient Positioning and Preparation

The patient is placed in the supine position on the operating table. After induction of regional anesthesia, a thorough knee examination is performed to assess ligamentous stability and range of motion. A well‐padded high‐thigh tourniquet is then applied to the operative leg.

### RF Tendon Harvest With Preserved Patellar Insertion and Graft Preparation

**FIGURE 2:**
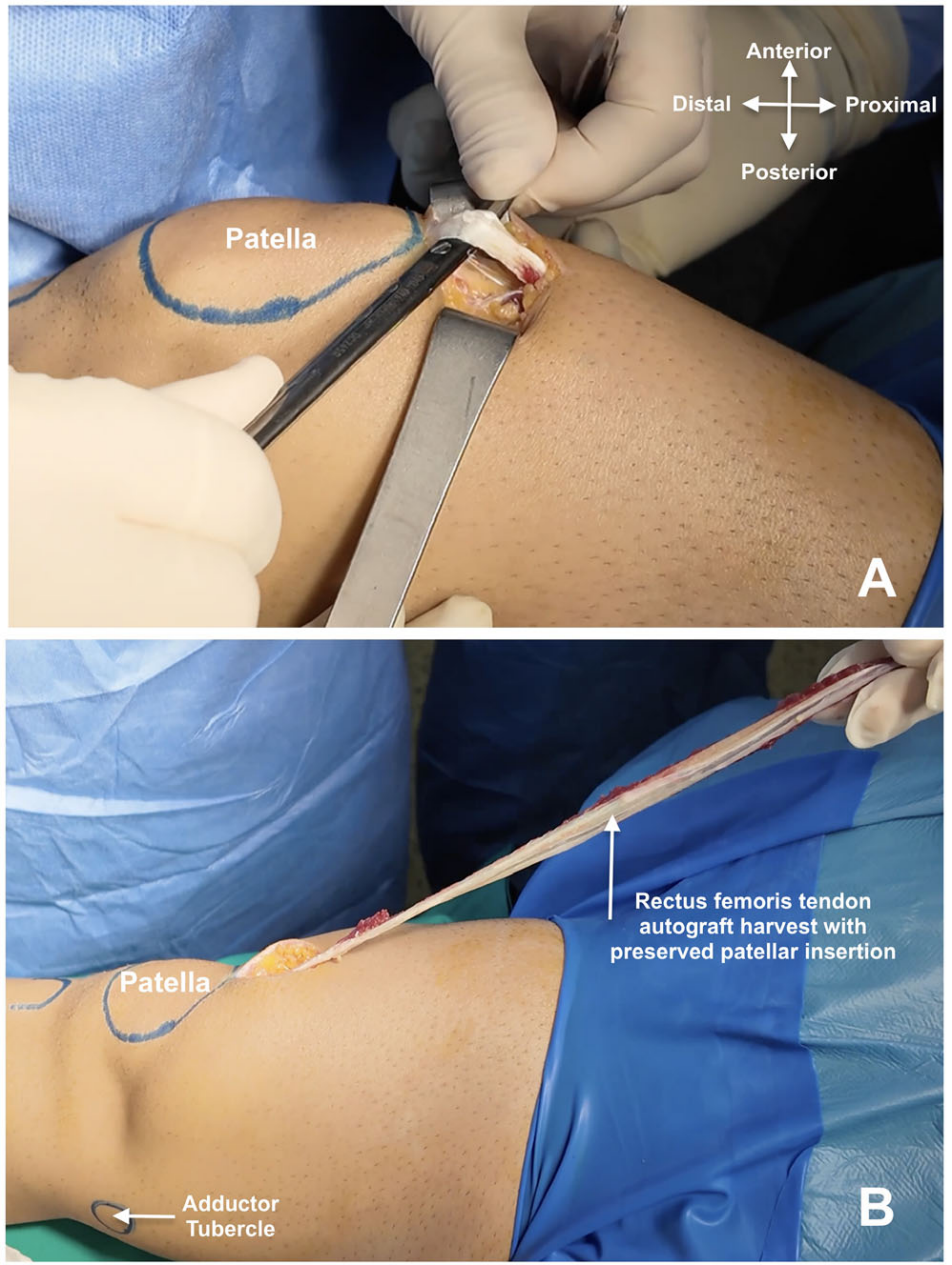
Right knee, patient in the supine position. Intraoperative harvest of the rectus femoris tendon. (A) Mobilization of the rectus femoris tendon using the fatty tissue layer, which separates the rectus femoris tendon from the vastus intermedius. (B) The harvested rectus femoris tendon graft, typically measuring 25 to 35 cm in length, with preservation of the distal patellar insertion.

A longitudinal skin incision of approximately 4 cm is made beginning 3 cm proximal to the superior border of the patella, positioned at the midline. The surgical plane between the superficial and intermediate laminae is identified approximately 3 cm proximal to the patella. After 2 longitudinal incisions on the RF tendon, an 8‐ to 10‐mm‐wide graft is fashioned. The fatty tissue layer is used to identify the plane between the superficial and intermediate laminae (Figure 2A). The RF tendon is elevated from the quadriceps tendon while preserving its distal patellar insertion. Proximal dissection of the tendon is then carried out for approximately 5 cm using scissors, ensuring that the intermediate and deep layers of the QT remain intact. With the knee flexed to 20°, an open‐ended tendon stripper is carefully advanced toward the anterior inferior iliac spine to complete harvest of the RF tendon graft. The harvested graft typically measures 25 to 35 cm in length, and residual muscle fibers are meticulously removed from the tendinous portion (Figure 2B).

### Femoral Socket Preparation

**FIGURE 3:**
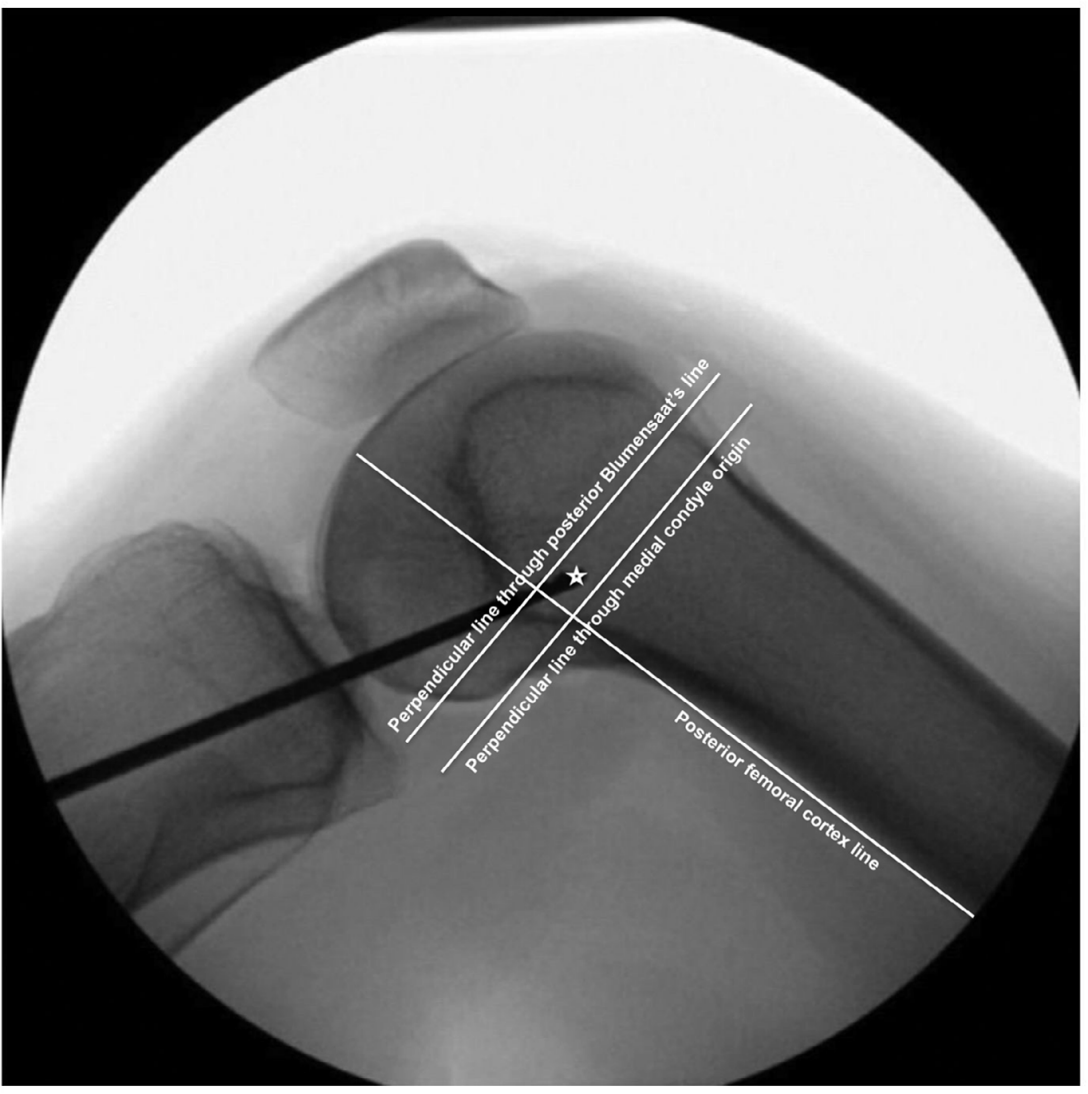
Intraoperative fluoroscopic images of the right knee. Radiographic determination of the femoral fixation site. The Schottle point (asterisk) is located anterior to the posterior cortex line and between the perpendicular medial condyle line and the perpendicular posterior Blumensaat line.

The anatomic femoral footprint is identified as a palpable bony landmark, typically located approximately 1 cm distal to the adductor tubercle, posterior to the medial epicondyle, and anterior to the gastrocnemius tubercle. At this location, a guide pin is introduced and directed anteriorly and proximally to avoid penetration of the joint line and potential injury to the posterior neurovascular structures. The pin placement is performed using anatomic landmarks and subsequently confirmed under fluoroscopy. A true lateral radiograph is obtained to ensure accurate positioning. The femoral fixation site is determined at the Schottle point, which lies 4 mm anterior to the posterior cortical line, distal to the posterior condylar origin, and proximal to the Blumensaat line (Figure 3). Once the position is confirmed, a femoral tunnel measuring 35 mm in length is drilled with a 7‐mm‐diameter reamer, and a passing pin is used to shuttle a No. 1 PDS suture through the tunnel, which will later serve to guide graft passage.

### Graft Rerouting, Passage, and Fixation

**FIGURE 4:**
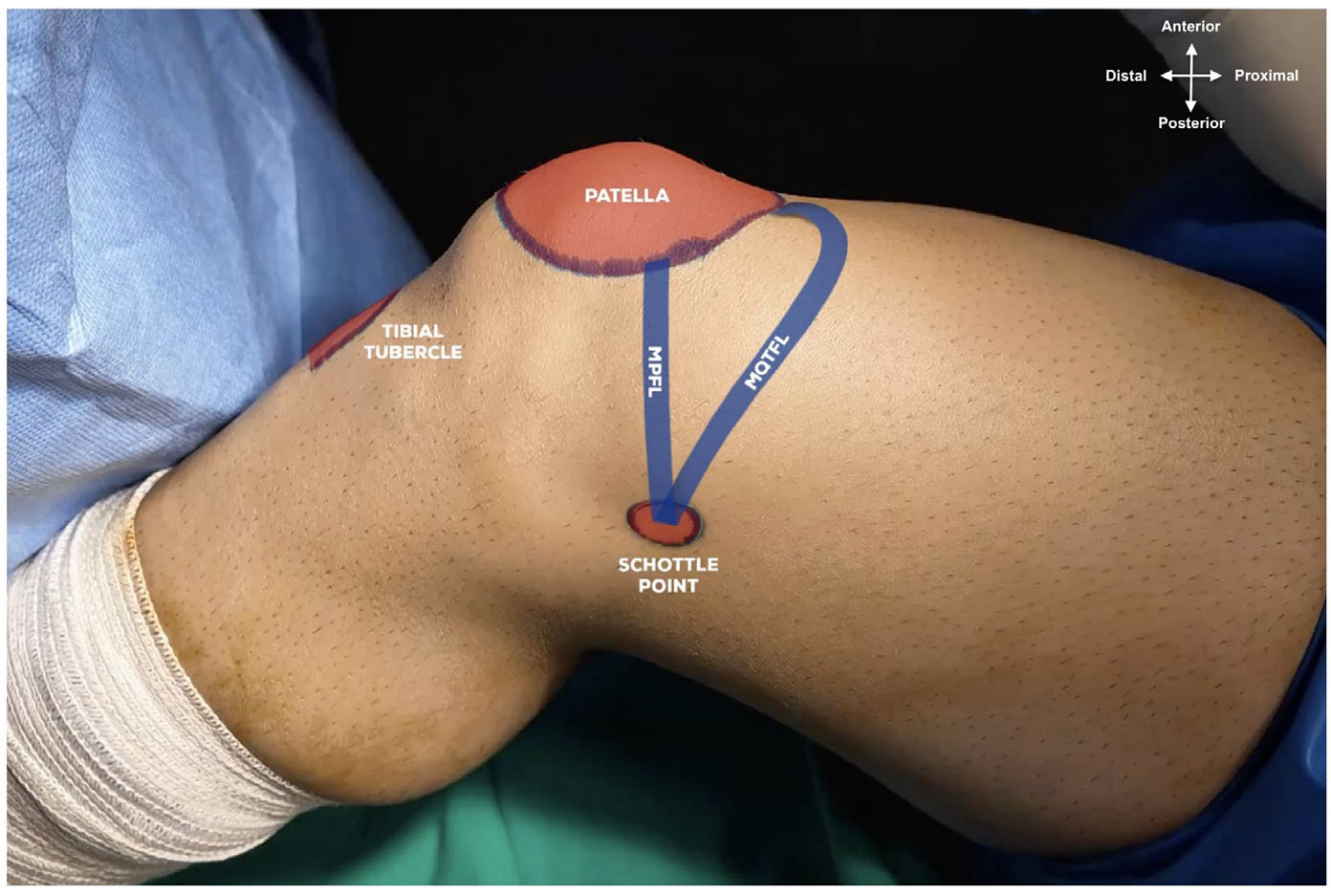
Right knee, schematic representation of graft rerouting. Rectus femoris autograft harvested with preserved patellar insertion, rerouted medially to reconstruct the MQTFL. The remaining limb is directed to the patella to recreate the MPFL, forming a V‐shaped medial patellofemoral complex. (MPFL, medial patellofemoral ligament; MQTFL, medial quadriceps tendon–femoral ligament.)

The RF autograft, harvested with its patellar insertion preserved, is first rerouted medially toward the femoral tunnel to reconstruct the MQTFL, and the remaining limb of the graft is then directed to the patella to recreate the MPFL, thereby forming a V‐shaped configuration of the MPFC (Figure 4).

**FIGURE 5:**
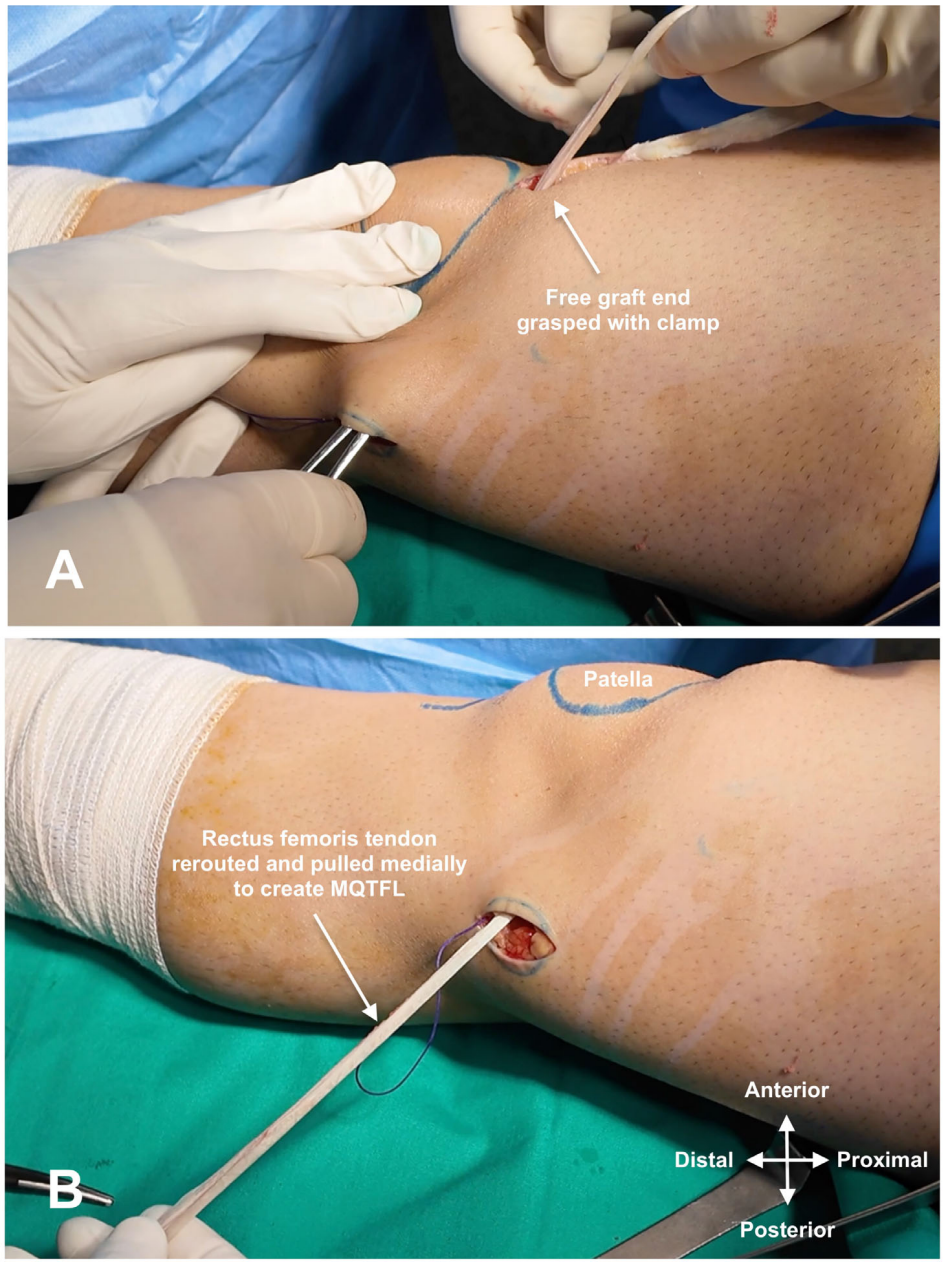
Right knee, intraoperative views. (A) The rectus femoris graft passed medially beneath the vastus medialis oblique using a clamp. (B) The rectus femoris graft rerouted and pulled medially to reconstruct the MQTFL. (MQTFL, medial quadriceps tendon–femoral ligament.)

**FIGURE 6:**
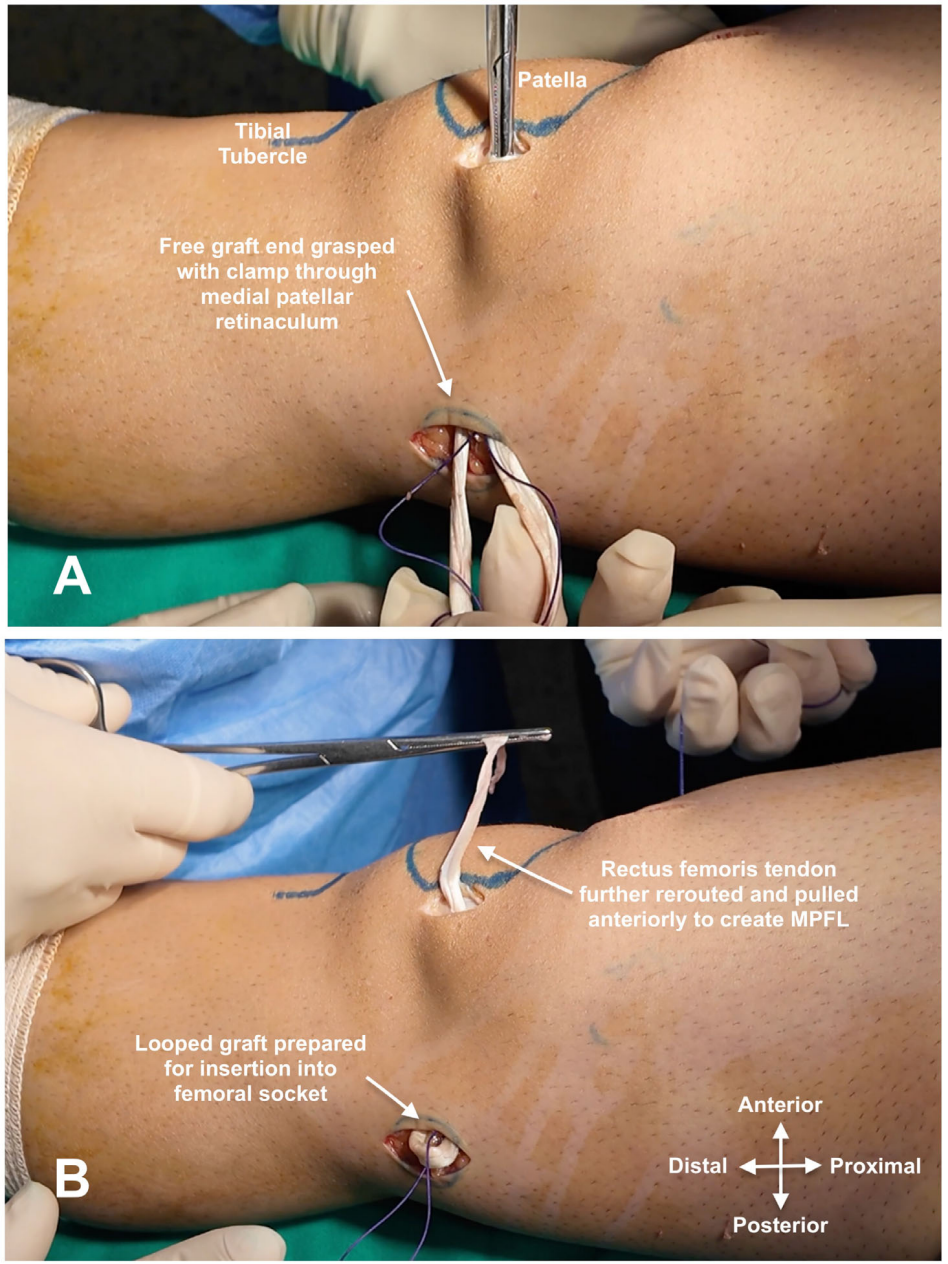
Right knee, intraoperative passage of the MPFL limb. (A) For the MPFL limb, the free end of the rectus femoris graft is grasped with a clamp and guided through the second soft‐tissue channel extending from the medial epicondyle to the medial patellar retinaculum. (B) The looped configuration of the rectus femoris graft is prepared for femoral fixation and positioned for insertion into the femoral socket with the aid of a shuttle suture. (MPFL, medial patellofemoral ligament.)

For the MQTFL limb, the first soft‐tissue channel is created between layers 2 and 3, extending from the adductor tubercle to the distal quadriceps tendon. The RF graft is passed medially beneath the vastus medialis oblique (VMO) muscle using a Kelly clamp, which grasps the graft near its tip to facilitate passage (Figure 5A,5B). For the MPFL limb, a separate skin incision is made just medial to the patella, and a second soft‐tissue channel is created from the medial epicondyle to the midportion of the patella (Figure 6A,6B).

**FIGURE 7:**
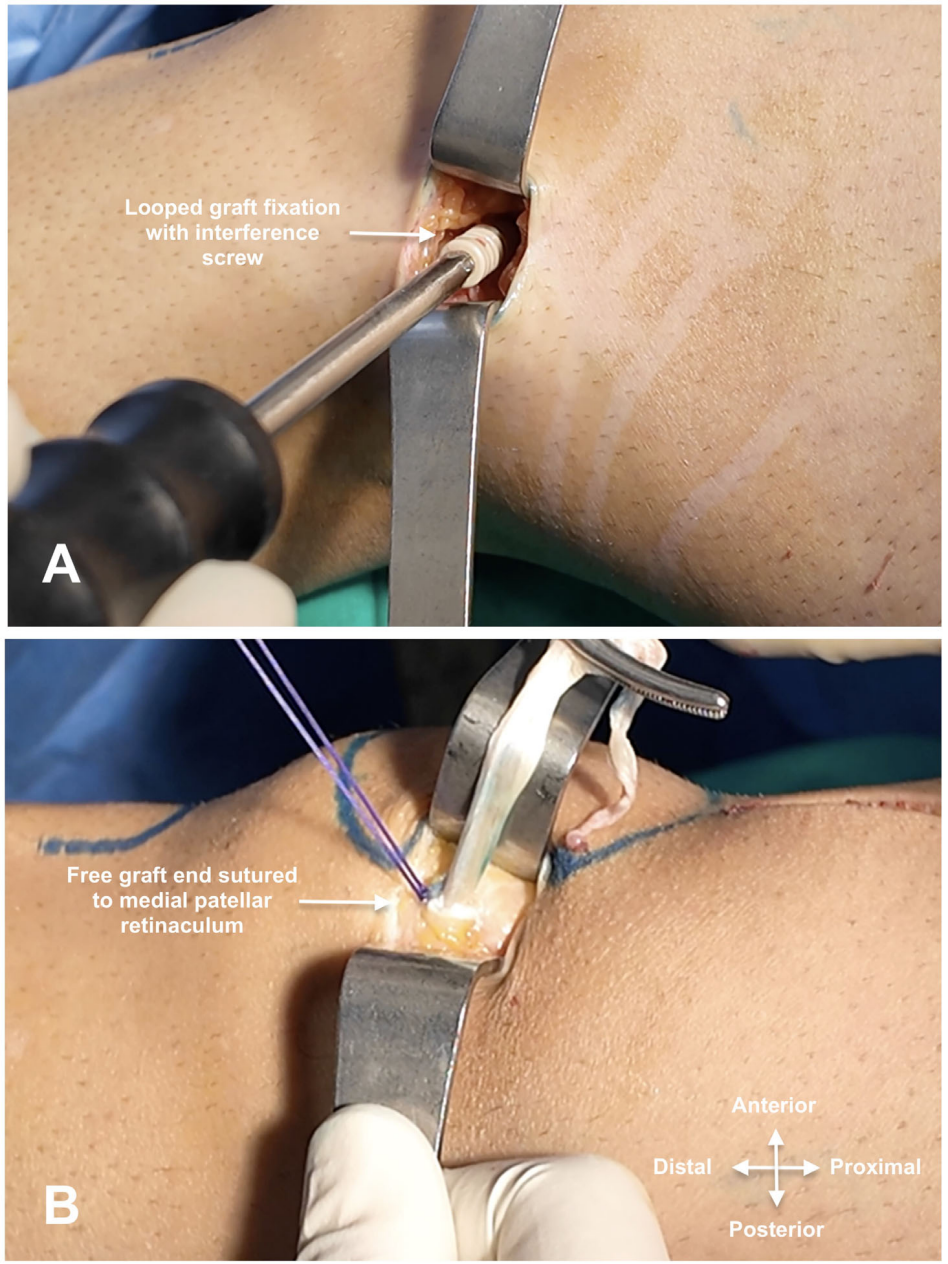
Right knee, intraoperative fixation steps. (A) Looped rectus femoris graft advanced into femoral socket and fixed with 8‐mm interference screw over a nitinol wire, completing reconstruct the medial quadriceps tendon–femoral ligament. (B) Remaining graft limb sutured to medial patellar retinaculum to reconstruct the medial patellofemoral ligament.

The graft is shuttled through the PDS passing suture and advanced into the femoral socket, where it is secured with an 8‐mm interference screw (Artroline, Turkey) over a nitinol wire, thus completing the MQTFL reconstruction (Figure 7A). The remaining limb of the graft is then directed to the patella and fixed to the medial patellar retinaculum using No. 2 nonabsorbable sutures, thereby recreating the MPFL (Figure 7B). In this manner, the anatomic MPFC is fully reconstructed.

**FIGURE 8:**
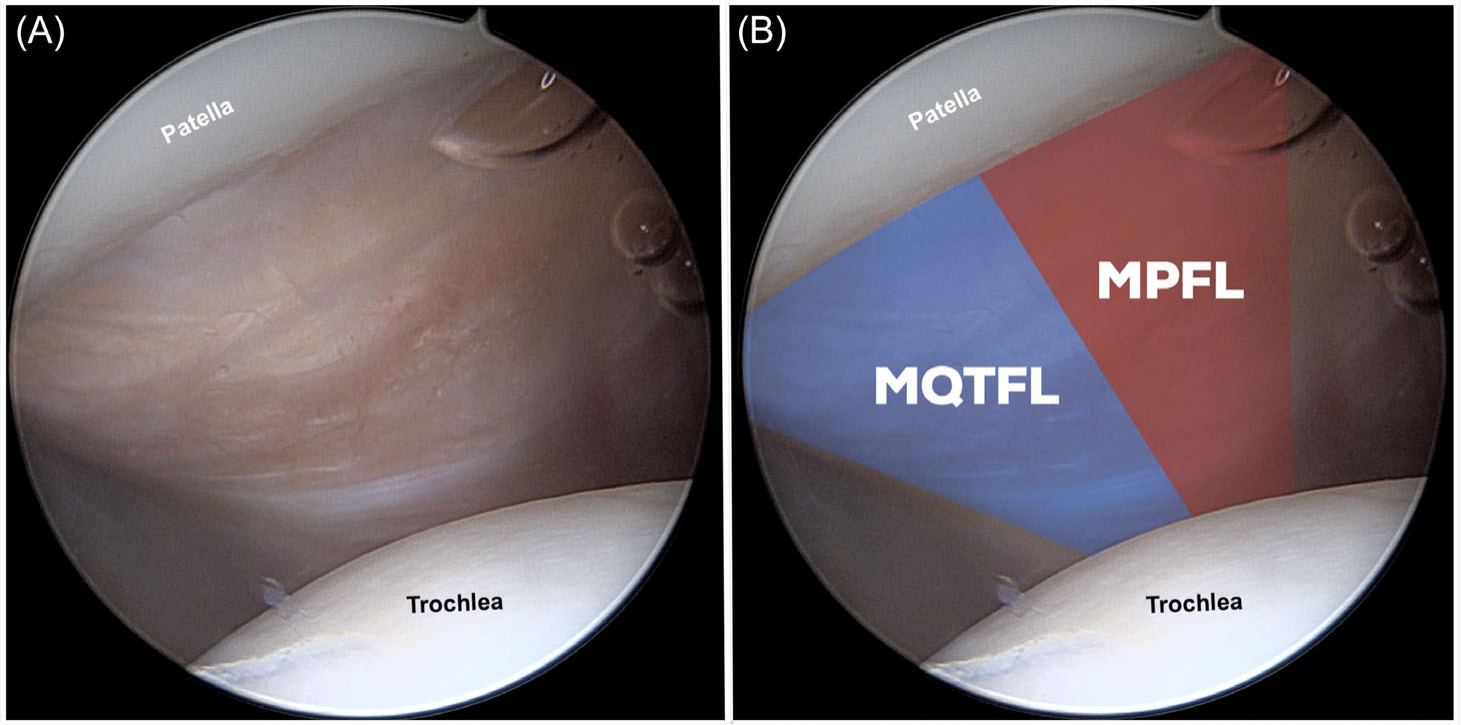
Right knee, supine position, arthroscopic views from the anterolateral portal. (A) Diagnostic arthroscopy, MPFC reconstruction to confirm extra‐articular graft positioning. (B) Both limbs of the rectus femoris graft are utilized separately to reconstruct the MQTFL and the MPFL, together forming a V‐shaped MPFC. (MPFC, medial patellofemoral complex; MPFL, medial patellofemoral ligament; MQTFL, medial quadriceps tendon–femoral ligament.)

A final arthroscopic examination is performed to confirm patellar stability, tracking, and appropriate graft tension (Figure 8A,8B). The tourniquet is then released, meticulous hemostasis is obtained, and all wounds are thoroughly irrigated and closed in layers. Finally, an elastic compression stocking is applied to reduce postoperative swelling.

### Rehabilitation

Immediate range‐of‐motion exercises and partial weight‐bearing with crutches are initiated and continued for the first 6 weeks. Thereafter, progressive physical therapy is advanced to full weight‐bearing, with patients permitted to resume light daily activities as tolerated. Return to sports is not recommended until at least 4 months postoperatively.

## DISCUSSION

Recognition of evolving medial patellofemoral anatomy has led to paradigm shifts in reconstructive approaches.^7^ While isolated MPFL reconstruction has historically been the mainstay of surgical treatment for recurrent patellar instability, recent anatomic studies have clarified that the MPFC consists of not only the MPFL but also the MQTFL.^5^, ^6^ This broader understanding emphasizes that restoring the MPFC, rather than a single ligament, provides a more accurate reproduction of native biomechanics.^11^ In this context, combined MPFL and MQTFL reconstruction respects the layered anatomy of the medial restraints and more closely replicates physiologic restraint across the range of knee motion.^7^, ^11^

Recent anatomical investigations have highlighted the variability of the MQTFL and its complex relationship with the MPFL.^5^ Fulkerson and Edgar^6^ originally described the proximal quadriceps fibers as a distinct structure, whereas other studies have considered these fibers part of a common condensation originating from the medial epicondyle. Cadaveric data by Tanaka et al.^5^ showed that the attachment sites of these fibers vary considerably, with some specimens attaching exclusively to the patella and others solely to the quadriceps tendon. Kang et al.^12^ further distinguished inferior‐straight fibers inserting on the patella from superior‐oblique fibers attaching to the quadriceps tendon, noting anisometry between the bundles throughout knee motion. These findings explain the variability in anatomic reports and underscore the importance of considering both patellar and quadriceps attachments during reconstruction. Biomechanical studies comparing single‐ and double‐bundle configurations, including Y‐ and C‐shaped techniques, have shown that double‐bundle reconstructions may better resist lateral patellar displacement in early flexion, although long‐term differences remain uncertain.^11^ Taken together, these observations emphasize that accurate restoration of the MQTFL, in addition to the MPFL, is critical for achieving an anatomic and functional reconstruction of the proximal medial patellar restraints.^11^ Our described technique, using an RF autograft with preserved patellar insertion, achieves an anatomic reconstruction in a V‐shaped configuration, recreating both the MPFL and MQTFL while avoiding patellar tunnels. We summarize the advantages and disadvantages in Table 1 and the pearls and pitfalls of our technique in Table 2.

**TABLE 1:** Advantages and Disadvantages

Advantages
• No implant required for fixation on the extensor mechanism
• Reduced overall cost due to minimal implant usage
• Avoids patellar tunnels, thereby eliminating risk of patellar fracture
• Preserves the native patellar insertion of the rectus femoris tendon, enhancing anatomic reconstruction
• Provides a V‐shaped configuration that restores both MPFL and MQTFL anatomy
• Suitable for revision MPFL cases

Disadvantages
• Harvesting the RF tendon while preserving its patellar insertion requires technical precision and carries a steeper learning curve
• Harvesting the RF tendon may weaken extensor mechanism and limit its future use for ACL grafts
• Potential donor site morbidity at the quadriceps tendon, such as anterior knee pain or extensor weakness or quadriceps inhibition

ACL, anterior cruciate ligament; MPFL, medial patellofemoral ligament; MQTFL, medial quadriceps tendon–femoral ligament; RF, rectus femoris.

**TABLE 2:** Pearls and Pitfalls

Pearls
• Make the harvesting incision 3 cm proximal to the superior pole of the patella to better identify the interval between the superficial and intermediate laminae of the quadriceps tendon (QT)
• Preserve the patellar insertion of the rectus femoris tendon during harvest to maintain anatomic continuity and facilitate V‐shaped MPFC reconstruction
• Strip the graft toward the anterior inferior iliac spine (AIIS) with the knee in 20° of flexion to relax the quadriceps
• Visualize layers 2 and 3 from the anterior incision to guide proper graft passage
• After graft passage, confirm position arthroscopically to ensure it has not penetrated layer 3 and entered the joint
• Confirm femoral tunnel placement using anatomic and radiographic landmarks, as well as intraoperative isometry, prior to reaming and fixation
• Tension the graft by removing slack with the knee at 60° of flexion to avoid overconstraint

Pitfalls
• During femoris harvesting, placing the incision too distal makes identification of the laminae impossible, as they merge together in this region
• Staying too medial increases the risk of harvesting a short and thin graft
• The graft may inadvertently be passed subcutaneously or intra‐articularly instead of between layers 2 and 3
• Inappropriate femoral tunnel positioning can negatively affect graft function
• Overtensioning of the graft can increase patellofemoral contact pressures and lead to pain

MPFC, medial patellofemoral complex.

In conclusion, combined MPFL and MQTFL reconstruction using an RF tendon autograft provides a more anatomic approach that respects the layered structure of the medial restraints and restores physiologic stability across knee motion. The use of an RF autograft with preserved patellar insertion offers a drill‐ and implant‐sparing technique that reduces the risk of fracture‐related complications. Future clinical studies are warranted to confirm long‐term outcomes and functional benefits of this V‐shaped MPFC reconstruction.

## DISCLOSURES

The authors (L.K., J.P.F., A.E.O.) declare that they have no known competing financial interests or personal relationships that could have appeared to influence the work reported in this article.

## INFORMED CONSENT

Informed consent was obtained from patient to use the patient information.

## CONSENT TO PARTICIPATE

The informed consent form, permission to participate in this clinical study, and the publication of the data of this study in any journal were obtained from a participant included in this study.
